# The relation between structural and functional connectivity depends on age and on task goals

**DOI:** 10.3389/fnhum.2014.00307

**Published:** 2014-05-16

**Authors:** Jaclyn H. Ford, Elizabeth A. Kensinger

**Affiliations:** Department of Psychology, Boston CollegeChestnut Hill, MA, USA

**Keywords:** diffusion weighted imaging, functional MRI, aging, emotion, prefrontal cortex, amygdala

## Abstract

The last decade has seen an increase in neuroimaging studies examining structural (i.e., structural integrity of white matter tracts) and functional connectivity (e.g., correlations in neural activity throughout the brain). Although structural and functional connectivity changes have often been measured independently, examining the relation between these two measures is critical to understanding the specific function of neural networks and the ways they may differ across tasks and individuals. The current study addressed this question by examining the effect of age (treated as a continuous variable) and emotional valence on the relation between functional and structural connectivity. As prior studies have suggested that prefrontal regions may guide and regulate emotional memory search via functional connections with the amygdala, the current analysis focused on functional connectivity between the left amygdala and the left prefrontal cortex, and structural integrity of the uncinate fasciculus, a white matter tract connecting prefrontal and temporal regions. Participants took part in a scanned retrieval task in which they recalled positive, negative, and neutral images associated with neutral titles. Aging was associated with a significant increase in the relation between measures of structural integrity (specifically, fractional anisotropy, or FA) along the uncinate fasciculus and functional connectivity between the left ventral prefrontal cortex and amygdala during positive event retrieval, but not negative or neutral retrieval. Notably, during negative event retrieval, age was linked to stronger structure-function relations between the amygdala and the dorsal anterior cingulate cortex, such that increased structural integrity predicted stronger negative functional connectivity in older adults only. These findings suggest that young and older adults may utilize a structural pathway to engage different retrieval and regulatory strategies, even when structural integrity along that pathway does not differ.

## Introduction

Over the last decade, neuroimaging researchers have undertaken the enormous task of understanding and mapping the human connectome (Bullmore and Sporns, 2009). Such studies typically examine two types of networks: structural networks representing the structural integrity of white matter tracts in the brain and functional networks reflecting the ability of two or more brain regions to interact with one another. Although structural (white matter) changes and functional (network activation) connectivity changes have often been measured independently, examining the relation between these two measures is critical to understanding the specific cognitive function of neural connections and the ways in which such functions may differ across tasks and individuals. The current paper will focus on these relations in human subjects, although these questions can also be addressed in animals using techniques not employed in humans.

A number of recent studies have revealed a strong relation between structural integrity and functional connectivity in resting state networks (see review by Damoiseaux and Greicius, 2009). Critically, however, there is not a direct one-to-one mapping between structural and functional connectivity. For instance, regions can be co-activated even when there is not a direct structural connection between the regions (Koch et al., 2002; Greicius et al., 2009; Honey et al., 2009), perhaps reflecting the ability for cognitive goals to moderate activity levels along pathways involving more than one node. Given the potential role of task goals in altering the relation between functional and structural connectivity, it is important to consider how variations in such goals across task conditions can alter the relation between these measures.

The relation between structural integrity and task-related functional connectivity may also vary as a function of healthy aging across the adult lifespan. Typical aging is associated with a number of neural changes, including changes in structural connectivity (e.g., O'Sullivan et al., 2001; Pfefferbaum and Sullivan, 2003; Madden et al., 2004). Importantly, healthy aging is not associated with decreased integrity of all white matter tracts. Age differences are greater in anterior relative to posterior tracts (e.g., Salat et al., 2005; Madden et al., 2009), and superior relative to inferior tracts (e.g., Zahr et al., 2009; Sullivan et al., 2010). In addition, greater age-related differences have been identified in anterior segments of white matter tracts, with differences increasing linearly from posterior to anterior segments (Davis et al., 2009). Similarly, age-related changes in functional connectivity have been demonstrated within resting state networks (e.g., Andrews-Hanna et al., 2007; Sambataro et al., 2010; Tomasi and Volkow, 2012; Ferreira and Busatto, 2013;) and task networks (e.g., St. Jacques et al., 2009a). Additionally, strong structural-functional connectivity relations in resting-state networks have been demonstrated in older adults (Andrews-Hanna et al., 2007), and left-right PFC functional connectivity is more strongly related to white matter integrity in the anterior corpus callosum (i.e., genu) within older relative to young adults (Davis et al., 2012).

Importantly, additional research has suggested that age-related differences in functional connectivity may differ depending on task demands. For instance, older adults show enhanced functional connectivity between the prefrontal cortex and the amygdala and hippocampus during successful encoding of positive relative to younger adults (Addis et al., 2010). While older age may be associated with *increased* prefrontal-amygdala functional connectivity while encoding positive information, it has been related to *decreased* functional connectivity between PFC and amygdala during successful encoding of negative information (St. Jacques et al., 2009a). Indeed, a review of age-related changes to neural activity during processing of negative information suggested that the two most prominent age-related differences were increased PFC activity and decreased amygdala activity (St. Jacques et al., 2009b).

These studies suggest that functional connectivity between PFC regions and medial temporal lobe (MTL) regions may be particularly affected by age-by-valence interactions during memory encoding.

The studies reviewed above suggest that the relation between functional and structural connectivity may depend on both task-related factors as well as individual differences. The overarching goal of the present study is to examine how the task-related factor of emotional valence and the individual-difference factor of age affect this relationship. Specifically, the current study examines the effect of age (as a continuous variable) on functional connectivity during retrieval of positive, negative, and neutral information (using psychophysiological analysis of fMRI), structural integrity (using DTI), and the relation between these two connectivity measures.

Prior studies have suggested that prefrontal-amygdalar connections may be particularly important for emotional memory retrieval, perhaps because prefrontal regions participate in the guidance of memory search and in the regulation of emotion (Ochsner and Gross, 2005), yet to date, most research has focused on prefrontal-amygdala connections during encoding of emotional information (St. Jacques et al., 2009a; Addis et al., 2010). As such, the current study focused specifically on connectivity between PFC and amygdala regions during emotional memory retrieval. A primary white matter tract associated with the limbic system, including the amygdala, is the uncinate fasciculus (UF; Von Der Heide et al., 2013), a tract associated with memory performance in a recent study (Schott et al., 2011). Previous functional connectivity analyses with the sample included in the current study identified significant age-related changes to prefrontal connectivity with the left medial temporal lobe (Ford et al., under review), leading to a focus in the current analysis on the left UF. Based on prior research focusing on structural-functional connectivity relations in another white matter tract (Davis et al., 2012), we hypothesize that such relations in the UF will increase as a function of age in the current study. In addition, we predict that these interactions will vary across task conditions (i.e., valence), reflecting age-by-valence interactions in functional connectivity.

## Methods

### Participants

Data from 55 healthy adults (mean age = 47.72, *SD* = 20.41, ages 19–85; mean education = 16.62, *SD* = 2.38; 23 female) are reported. Twenty-seven of the young adult subjects (ages 18–39) from this sample were included in a recent paper examining the interactive effects of emotional valence and memory phase on neural recruitment (Ford et al., 2014). The participants in the current study are a subset of those reported in another analysis from our lab (Ford et al., under review). Gender distribution was even across the age range and age was not significantly correlated with education (*p* = 0.87). Two additional participants were recruited but not scanned due to contraindications for fMRI (ages 50 and 75; both male). Another twelve participants were scanned, but were excluded from the current analysis due to equipment malfunction (*n* = 1; age = 49, edu = 16, male), an abnormal structural scan (*n* = 1, age = 49, edu = 17, female), early termination of the MR session due to excessive motion (*n* = 1, age = 56, edu = 16, male), voluntary early termination of the MR session (*n* = 1, age = 49, edu = 14, female), truncated medial temporal lobe activity due to signal drop out (*n* = 2, ages 23 and 58, both female) or low behavioral performance (i.e., below chance; *n* = 6, mean age = 55.64, *SD* = 18.12, ages 30–83; mean education = 16.12, *SD* = 3.49; 2 female). In addition, six participants had usable fMRI data, but had poor data quality that prevented analysis of diffusion data (mean age = 52.17, *SD* =13.99, ages 36–70; mean education = 16.33, *SD* = 1.86; 4 female). Participants were right-handed native English speakers without psychiatric illness or neurological disorder and were recruited from the greater Boston area. All participants were paid for their participation and gave written informed consent in accordance with the requirements of the Institutional Review Board at Boston College.

Movement during functional scans was examined using the ART toolbox (http://web.mit.edu/swg/software.htm) in SPM8. Using this toolbox, we identified scans in which motion was more than three standard deviations away from the mean, scans that were at least 5 mm from the starting location, and scans that exhibited more than 0.05° rotation in any plane. No subjects were excluded based on this analysis, as no one had more than 4 time points that met any of these criteria in any condition of interest, and a majority of participants had 1 or fewer (*M* = 0.25 outliers per participant in the neutral event condition, *M* = 0.36 outliers per participant in the positive event condition, and *M* = 0.29 outliers per participant in the negative event condition). Importantly, age was not associated with the number of outliers (*p* = 0.71) and number of outliers did not differ across emotion conditions (*p* = 0.23). Additionally, there was no interactive effect of age and valence on the number of outliers (*p* = 0.19).

All participants completed the Beck Anxiety Inventory (Beck et al., 1988) to examine self-reported symptoms of anxiety, as well as the Beck Depression Inventory (Beck et al., 1961) and the Geriatric Depression Scale (Sheikh and Yesavage, 1986) to evaluate symptoms of depression. In addition, participants engaged in a series of tests intended to examine general cognitive ability, vocabulary, verbal fluency, working memory, and long-term memory (both immediate and delayed), and all participants completed a battery of cognitive tests implemented in CogState, a computerized neuropsychological test battery, that was approximately 30 min in duration. The battery included 6 subtests that examine a range of cognitive abilities, including: Detection Task (speed of processing), Identification Task (visual attention), One Card Learning Task (visual learning and memory), One Back Task (attention/working memory), Two Back Task (attention/working memory), and Set-Shifting (executive function); these have acceptable criterion and construct validity in a neuropsychological context (see www.cogstate.com; Maruff et al., 2009). The relations of age with all cognitive variables are reported in Table 1. In addition to being screened for dementia, that older adults in this sample have been screened for a number of other health problems often associated with increased age that can interfere with interpretation of the fMRI BOLD signal (e.g., high blood pressure not controlled by medication). As such, the findings in the current study are limited to *healthy* aging.

**Table 1:** **Correlations between age and cognitive tests from all 55 subjects, with averages and standard deviations from 12 youngest and 12 oldest participants**.

**Measure**	***r***	***p***	**Youngest participants**	**Oldest participants**	**Reference for measure**
Beck anxiety inventory	−0.37	0.005^*^	7.25 (5.64)	2.17 (2.66)	Beck et al., 1988
Beck depression index	0.01	0.944	3 (3.02)	2.67 (1.97)	Beck et al., 1961
Geriatric depression scale–short form	−0.14	0.318	0.9 (0.99)	0.25 (0.62)	Sheikh and Yesavage, 1986
Mini−mental state exam 2	−0.38	0.003^*^	29.5 (0.80)	28.33 (1.15)	Folstein et al., 1975
Shipley vocabulary	0.38	0.004^*^	33.33 (3.31)	37.25 (3.44)	Shipley, 1986
Generative naming	0.13	0.341	44.25 (11.27)	46.33 (12.56)	Spreen and Benton, 1977
**WECHSLER ADULT INTELLIGENCE SCALE (Wechsler, 1997a)**					
Digit symbol substitution–60 s	−0.52	0.000^*^	45.42 (7.28)	33 (6.34)	
Digit symbol substitution–90 s	−0.53	0.000^*^	68.92 (9.02)	52.27 (8.75)	
Mental arithmetic	−0.22	0.100	16.17 (3.33)	14.08 (3.15)	
Forward digit span	−0.30	**0.027**	12.42 (2.19)	10.73 (2.05)	
Backward digit span	−0.24	0.078	9.17 (2.48)	8.42 (2.19)	
**WECHSLER MEMORY SCALE (Wechsler, 1997b)**					
Logical memory–immediate	0.09	0.504	30.42 (8.04)	31.25 (5.28)	
Logical memory–delayed	0.00	0.978	32.42 (9.52)	31.08 (7.50)	
Logical memory–recognition	0.08	0.545	27.33 (2.57)	27.75 (1.48)	
Verbal pairs–immediate	−0.24	0.075	24.42 (6.68)	21.33 (7.00)	
Verbal pairs–delayed	−0.12	0.393	7 (1.60)	6.5 (2.11)	
Visual pairs–immediate	−0.21	0.122	16.75 (1.29)	13.92 (3.75)	
Visual pairs–delayed	−0.38	0.004^*^	6 (0.00)	5.08 (1.44)	
Mental control	−0.32	0.015^*^	30.75 (5.08)	26 (4.75)	
**COGSTATE RESEARCH BATTERY (Maruff et al., 2009; Pietrzak et al., 2009)**					
Set shifting–speed	0.69	0.000^*^	2.69 (0.19)	3.22 (0.23)	
Set shifting–accuracy	−0.42	0.001^*^	84.9 (6.54)	74.4 (9.11)	
Detection–speed	0.38	0.005^*^	351.1 (87.15)	536.33 (144.60)	
Detection–accuracy	−0.13	0.361	107.18 (32.81)	98.16 (3.87)	
Identification–speed	0.25	0.068	465.8 (85.99)	645.92 (111.95)	
Identification–accuracy	0.13	0.352	95.83 (4.93)	95.78 (4.83)	
One card learning–speed	0.39	0.003^*^	916.8 (129.78)	1242.17 (304.74)	
One card learning–accuracy	−0.22	0.109	69.1 (4.14)	67.36 (12.35)	
One-back–speed	0.52	0.000^*^	672.2 (138)	1048.92 (224.91)	
One-back–accuracy	−0.24	0.079	107.2 (8.93)	103.58 (12.21)	
Two-back–speed	−0.13	0.343	2.87 (0.12)	3.1 (0.11)	
Two-back–accuracy	−0.22	0.102	93.48 (5.03)	87.29 (7.06)	

*All values represent raw, non-standardized means with standard deviations in parentheses*.

*Significant age-related differences are bolded*.

* *Contrast survived False Discovery Rate correction*.

### Behavioral task materials

Stimuli were the 480 pictures (160 positive, 160 negative, and 160 neutral) and the neutral titles used in Ford et al. (2014). Based on normative data available at the time of image selection, arousal ratings were equated for positive and negative images, while positive and negative images were significantly higher in arousal than neutral images. However, arousal ratings given by participants in the current study were higher for negative relative to positive images (see behavioral results section). The 480 title-picture pairs were divided into four sets of 120 pictures each (40 positive, 40 negative, and 40 neutral) for counterbalancing purposes. Arousal ratings for positive (*p* = 0.29) and negative (*p* = 0.97) images did not differ across the four sets of pictures.

### Behavioral task procedure

Following instruction and a short practice, participants encoded one set of 120 title-image pairs. Titles (e.g., “Lettuce”) were paired with a positive, negative, or neutral image (e.g., a piece of rotting lettuce with bugs crawling on it as a negative image). In an intentional encoding task participants were given 3 s to make a decision regarding the appropriateness of the word as a description of the image (1 = poor description, 2 = acceptable description, and 3 = very good description). After a half-hour delay (*M* = 34.3 min, *SD* = 7.8), participants took part in a scanned retrieval task. Participants were presented with the 240 titles (120 neutral titles that were studied during the encoding phase and 120 unstudied neutral titles) randomly across 6 retrieval runs of equal length. Participants were given up to 4 s to decide whether the word was “old” (i.e., seen previously) or “new” (i.e., not seen previously). The screen was removed following the participant's button press. Across participants, it was varied which items were studied and which were reserved as foils on the recognition test.

Immediately following an “old” response, 80% of the time, participants were asked to “Elaborate” on the old item (i.e., think about the image presented with the title and the experience with that title and image at encoding) for 5 s. Participants were then presented with two ratings that asked them to consider the vividness of their memory on a 1–5 scale. In the first, they were asked to rate how well they remembered the image itself; in the second they rated how well they remembered their own personal thoughts and feelings while encoding the item. Each rating was presented for 3 s and the order of the ratings was alternated across participants. To discourage participants from beginning to elaborate during the search phase, and to distinguish activity during search from activity during elaboration, 20% of trials were catch trials; instead of an elaboration phase, the next trial was presented. Following a “new” response, 80% of the time, participants moved on to the next trial. To minimize the likelihood that participants would automatically begin preparing for the next trial after a “new” response, on 20% of the trials, participants were asked to “Imagine” an image that could have accompanied the new item for 5 sec. They then performed two ratings: They rated (on a 1–5 scale) the vividness of the image they generated for the new item and the vividness of their own personal thoughts and feelings. Following each trial, participants viewed a fixation cross for 0–6 s to introduce jitter. The length and distribution of jitter durations was determined using the optseq software (http://surfer.nmr.mgh.harvard.edu/optseq/). However, as it is impossible to know when participants will answer correctly, the optimal order across conditions could not be utilized. Instead, the jitter durations were assigned to each trial randomly within our presentation software.

After being removed from the scanner, participants were re-presented with the images from the encoding phase. They rated each image's valence and arousal on a 1–7 scale and indicated which specific emotions they experienced with each image. This portion was self-paced, but participants were encouraged to respond based on their initial reaction.

### MR data acquisition

Participants' heads were stabilized in a Siemens Tim Trio 3 Tesla scanner. A localizing scan and auto-align scout were followed by a high resolution multi-echo T1 structural scan for anatomical visualization (176 1 mm slices, TR = 2200 ms, TE1 = 1.64 ms, TE2 = 3.5 ms, TE3 = 5.36 ms, TE4 = 7.22 ms). Six runs of whole brain, gradient-echo, echo planar images (31 3 mm slices aligned along the line between the anterior and posterior commissures, 20% skip, TR = 2 s, TE = 30 ms, Flip angle = 90) were acquired during memory retrieval using interleaved slice acquisition. Response data were collected using a magnet-safe button response box. Diffusion-weighted images were collected using a twice-refocused spin echo (Reese et al., 2003) DTI protocol (5 min 23 s total) that included 5 non-diffusion-weighted volumes (*b* = 0) and 30 diffusion weighted volumes acquired with non-colinear gradient directions (*b* = 700 s/mm^2^). Isotropic voxel resolution was 2.0 mm, base resolution 108 × 108 and 68 slices, employing TE/TR = 86/8450 ms, parallel imaging (GRAPPA) acceleration of 2, and 7/8 partial fourier.

### MR data preprocessing and analysis

The primary goal of the present study is to examine the relation between structural integrity and functional connectivity during positive, negative, and neutral event retrieval, and the effect of age on this relation. To this end, the current analysis was performed in three steps: (1) Preprocessing and analysis of diffusion data, (2) Preprocessing and analysis of functional connectivity data, using generalized psychophysiological interactions (gPPI) analysis (3) Analysis of the effects of age and emotion on the relation between Fractional Anisotropy (FA) and gPPI measures.

#### Preprocessing and analysis of diffusion data

**Figure 1:**
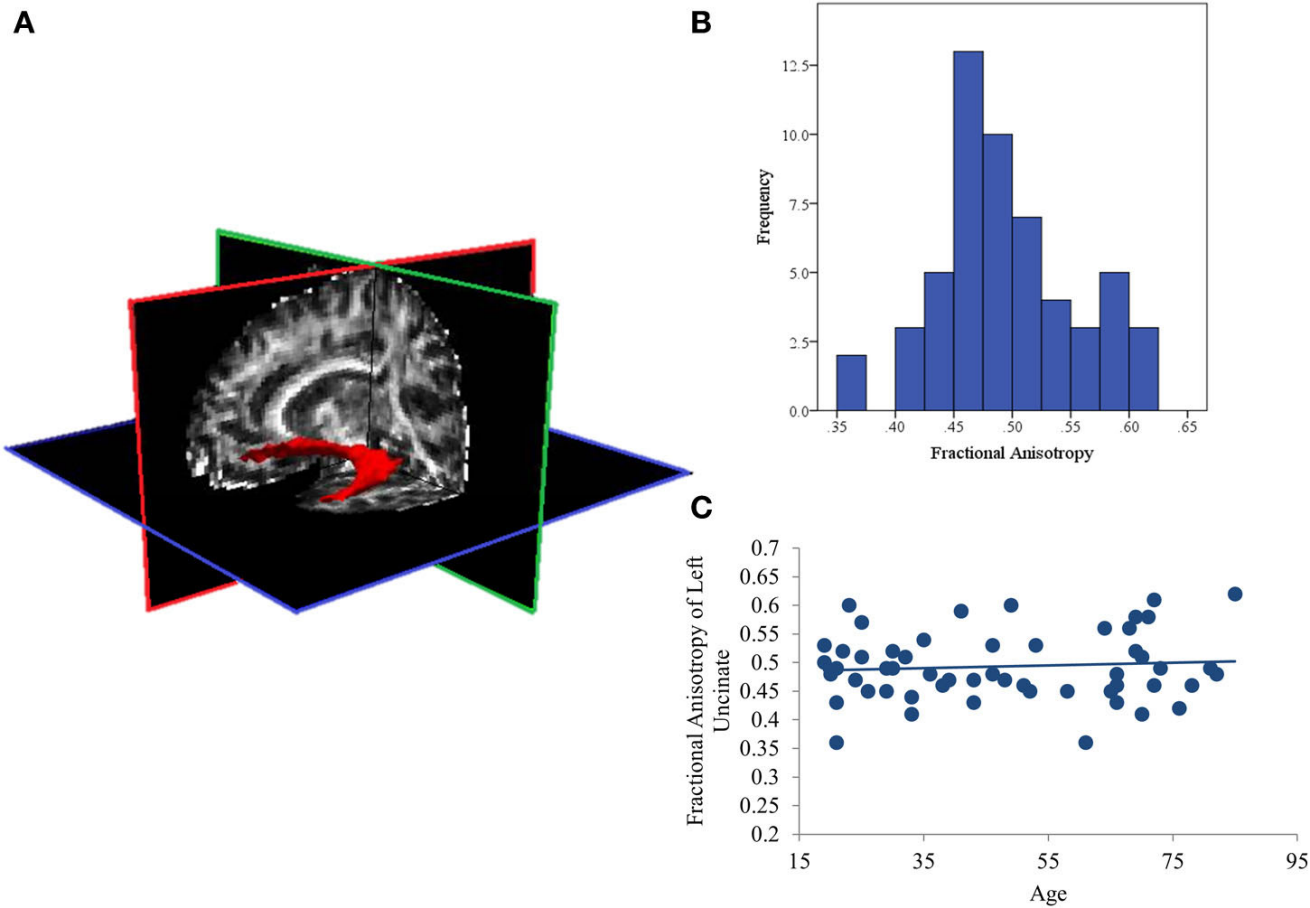
**(A)** Representative posterior distribution of the reconstructed uncinate fasciculus thresholded at 20% maximum; **(B)** Histogram of average Fractional Anisotropy (FA) values for the uncinate fasciculus in all subjects; **(C)** Scatterplot showing that aging is not associated with FA values in this sample.

Cortical reconstruction and volumetric segmentation of structural MRI data were performed using an automated processing stream in Freesurfer v5.1.0 (http://surfer.nmr.mgh.harvard.edu/; e.g., Dale et al., 1999; Fischl et al., 2001, 2002; Segonne et al., 2004; Han et al., 2006; Reuter et al., 2012). This stream includes motion correction and averaging, automated transformation, spatial smoothing, removal of non-brain tissue, segmentation of white and gray matter, and intensity normalization. We defined the white matter tract of interest (the uncinate fasciculus) using FreeSurfer's TRActs Constrained by UnderLying Anatomy (TRACULA; Yendiki et al., 2011), an automated method that reconstructs probabilistic distributions of major white matter tracts from each participant's diffusion images. This method has been shown to accurately reconstruct tracts in individual subjects using anatomical priors. TRACULA is made up of three processing steps: preprocessing, fitting of a ball-and-stick model of diffusion to the diffusion-weighted data, and reconstruction of the pathways of interest. TRACULA preprocessing includes eddy-current compensation, motion correction, intra-subject registration (to subject's T1), inter-subject registration (to MNI template), creation of cortical and white-matter masks, tensor fitting, and computation of anatomical prior for white-matter pathways. The ball-and-stick model is applied to each subject's preprocessed diffusion data, estimating parameters of the model at every voxel. These results, along with prior knowledge of pathway anatomy, are then used to fit the pathway of interest (here, the uncinate fasciculus, see Figure 1A for depiction of a representative pathway) for each subject. Measures of fractional anisotropy (FA; i.e., a ratio of radial and axial diffusivity) are then extracted from the estimated pathway.

#### Preprocessing and analysis of functional connectivity data

Functional MR Images were preprocessed and analyzed using SPM8 software (Wellcome Department of Cognitive Neurology, London, UK) implemented in MATLAB. Images were co-registered, realigned, normalized (resampled at 3 mm at the segmentation stage and written at 2 mm at the normalization stage) and smoothed using a Gaussian 8 mm kernel. Each memory trial included either an initial search phase followed immediately by the next trial (“old” catch trials and “new” non-catch trials) or an initial search phase followed by an elaboration phase (“old” non-catch trials and “new” catch trials).

At the individual subject level, a fixed effects model was created that included separate regressors of interest for accurate “old” responses to studied positive, negative, and neutral items (i.e., “hits”) and accurate “new” responses to unstudied positive, negative, and neutral items (i.e., “correct rejections”). Incorrect responses and time spent making vividness ratings, although not relevant for the current analysis, were included in each model as two separate nuisance variables. Each event of interest was modeled as a zero-duration event, convolved with the default Gaussian hemodynamic response function in SPM8. In the current analysis we were particularly focused on functional connectivity during the initial search phase and thus modeled a zero-duration event at the timepoint when the initial retrieval cue was presented; modeling in this way should limit the effects of variability in cognitive processes that may occur later in the retrieval trial.

**Figure 2:**
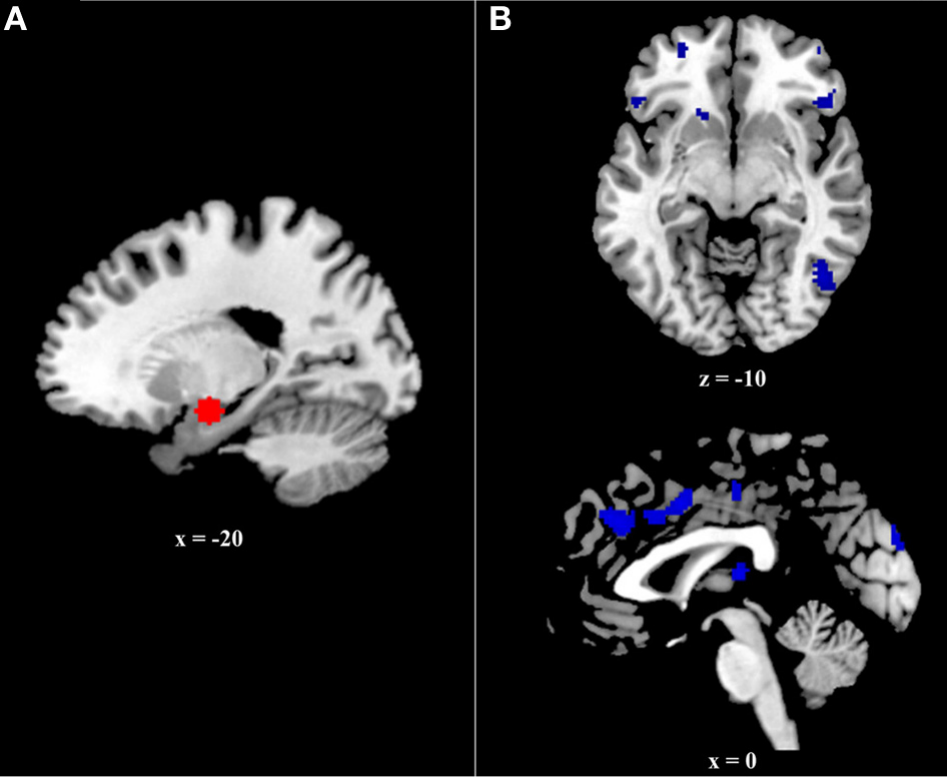
**(A)** Six millimeter sphere used as the volume of interest (VOI) for psychophysiological analysis. The center of this VOI (−20, −8, −16) was selected via an omnibus contrast that identified regions associated with retrieval (Hits > Correct Rejections) of all events (positive, negative, and neutral), controlling for age. VOIs were created and defined functionally at the single-subject level. **(B)** Regions exhibiting greater age effects on the relation between functional and structural connectivity during positive event retrieval relative to negative event retrieval.

The current study examined connectivity between the amygdala and prefrontal regions during successful retrieval of positive, negative, and neutral events, utilizing the generalized psychophysiological interactions (gPPI; http://brainmap.wisc.edu/PPI; McLaren et al., 2012) toolbox in SPM8. The gPPI toolbox, which is configured to automatically accommodate multiple task conditions in the same PPI model, compares functional connectivity to a single seed region across tasks. Due to an a priori interest in connectivity with the amygdala, it was selected as our seed region. To identify the voxel within the amygdala showing the greatest effect of memory (i.e., greatest BOLD response to Hits>CRs), we ran an omnibus contrast at the group level that identified regions associated with retrieval (Hits>Correct Rejections) of all events (positive, negative, and neutral), controlling for age. The peak voxel within the amygdala (−20, −8, −16) from this group contrast, was used to create volumes of interest (VOIs) for each subject. Specifically, for each subject, a VOI was generated by creating a 6 mm sphere around this voxel (Figure 2A). Within each subject, the gPPI toolbox was used to estimate functional connectivity across the entire brain with this 6 mm VOI in the six memory conditions (i.e., Positive hits, positive CRs, negative hits, negative CRs, neutral hits, and neutral CRs) and to calculate the three contrasts of interest (i.e., Positive Hits>CRs, Negative Hits>CRs, and Neutral Hits>CRs).

#### Analysis of the effects of age and emotion on the relation between FA and gPPI measures

A full factorial model was conducted at the random-effects level in which the three gPPI contrasts specified at the fixed-effects level (i.e., Positive Hits>CRs, Negative Hits>CRs, and Neutral Hits>CRs) were entered as three separate conditions with uncinate FA, Age, and the interaction of Age and FA as three covariates of interest. The current analysis was primarily interested in how age influenced the relation between functional and structural connectivity, and how this relation may be different as a function of emotional valence. As such, we performed a *t*-test within SPM8 comparing the interactive effect of age and FA in positive and negative event retrieval (i.e., Pos Age*FA > Neg Age*FA and Neg Age*FA > Pos Age*FA). These contrasts were evaluated at a *p* < 0.005 threshold with a 10 voxel extent, as this threshold has previously been shown to strike an appropriate balance between type 1 and type 2 error in fMRI analyses (Lieberman and Cunningham, 2009).

To understand the directionality of these effects, beta estimates of functional connectivity with the left amygdala were extracted from selected prefrontal regions of interest (ROIs) using the REX toolbox (downloaded from http://web.mit.edu/swg/software.htm) and these values were entered into a regression analysis with mean-centered age, mean-centered uncinate FA, and the interactive effect of age and uncinate FA, as predictors. Because mean-centering our age variable examines the effect of FA at age 47, it can only provide information regarding the effects of FA in middle-aged adults. Therefore, although the regression analysis is able to identify interactions, it cannot explain the reason for the interaction. To further interrogate significant interactions, additional models were created with each decade as the zero-point, providing a more complete representation of the interaction. These models were statistically identical to the mean-centered regression, aside from a different regression coefficient for the effect of FA on functional connectivity. This coefficient was used for visualization purposes to better understand the age by FA interaction. In addition, this approach allows for an approximate estimation of when along the lifespan the effect of FA becomes significant.

## Results

### Behavioral results

Memory accuracy and retrieval time were entered into a repeated measures ANOVA, with age as a covariate of interest and emotional valence (i.e., positive, negative, and neutral) as a within-subjects factor. Accuracy did not differ as a function of valence (*p* = 0.53), but was associated with age [*F*_(1,53)_ = 25.03, *p* < 0.001]. Specifically, increased age was associated with decreased accuracy for neutral (*r* = −0.47, *p* < 0.001), positive (*r* = −0.51, *p* < 0.001), and negative events (*r* = −0.52, *p* < 0.001). There was a trend for valence to affect retrieval time (*p* = 0.08), and aging was associated with increased retrieval times [*F*_(1, 63)_ = 10.45, *p* < 0.005] for neutral (*r* = 0.44, *p* < 0.001], positive (*r* = 0.40, *p* < 0.005), and negative events (*r* = 0.35, *p* < 0.01). Importantly, the age-by-valence interaction was not significant for either variable (*p* > 0.4 for both comparisons).

Ratings collected after the memory test confirmed that positive images were judged as more positive than neutral images [*t*_(54)_ = 10.85, *p* < 0.001] which were judged as more positive than negative images [*t*_(54)_ = 12.85, *p* < 0.001]. Additionally, negative and positive images were judged as more arousing than neutral images (*p* < 0.001 for both contrasts), and negative images were more arousing than positive [*t*_(54)_ = 3.19, *p* < 0.005]. Age was not associated with ratings of arousal or valence for any emotion condition (*p* > 0.3 for all contrasts).

### Effects of aging on white matter integrity of the uncinate fasciculus

The distribution of FA values was normal (See Figure 1B) with no significant outliers. In addition, aging was also not related to white matter integrity of the left UF. The current analysis found null effects of age on FA (*p* = 0.64; See Figure 1C). Similarly, comparing these measures of structural connectivity in the 19 youngest (ages 19–34; first tertile) and 18 oldest (ages 65–85; third tertile) participants also found no effect of age (*p* = 0.47).

### Effects of aging and emotion on the relation between white matter integrity and functional connectivity between the amygdala and the ventral PFC

The current analysis examined prefrontal regions in which the effect of FA on amygdalar functional connectivity differed as a function of age. More specifically, it compared this age by FA interaction during positive and negative event retrieval. The first contrast, Neg Age*FA > Pos Age*FA, revealed no significant clusters. Conversely, the second contrast, Pos Age*FA > Neg Age*FA, had a number of significant clusters, including ventral prefrontal cortex and dorsal anterior cingulate cortex (Table 2 and Figure 2B). Regions identified in this contrast can be driven by either a strong positive Age*FA effect during positive event retrieval or a strong negative Age*FA effect during negative event retrieval. In other words, aging may be associated with more a more positive relation between functional and structural connectivity during positive event retrieval or a more negative relation during negative event retrieval. To disambiguate these options, we examined contrast maps for Positive event retrieval > baseline and Negative event retrieval < baseline for significance of each peak in the interaction. If a peak was significant in one of these maps at *p* < 0.005, with a 10 voxel extent, this is indicated in Table 2.

**Table 2:** **Regions in which the effects of age on the relation between functional and structural connectivity was greater for positive relative to negative event retrieval**.

**MNI Coordinates**								
**Region of interest**	**Hemisphere**	**BA**	***x***	***y***	***z***	***t*-value**	***k***	**Interaction direction**
**FRONTAL**								
Premotor cortex	R	6	14	10	58	3.63	513	Negative
Dorsolateral prefrontal cortex	L	8	−28	18	44	3.24	70	Negative
	R	8	34	24	48	3.15	41	Negative
Ventrolateral prefrontal cortex	L	11	−40	36	−18	3.15	13	
	L	47	−50	26	−8	2.89	19	Positive
	L	47	−28	30	−16	2.88	14	Negative
	R	47	40	28	−4	3.12	123	Negative
**Ventral prefrontal cortex**	**L**	**11**	−**26**	**54**	−**10**	**3.08**	**12**	**Positive**
	R	10	42	56	−4	2.90	21	
Anterior prefrontal cortex	L	10	−28	62	8	2.77	15	Negative
**TEMPORAL**								
Fusiform gyrus	R	20	44	−26	−18	3.32	50	
Superior temporal gyrus	R	22	54	−54	12	2.93	75	
	R	22	54	6	4	2.92	42	Negative
**PARIETAL**								
Postcentral gyrus	L	40	−60	−20	22	4.32	540	Negative
Supramarginal gyrus	R	40	50	−42	32	3.94	166	Positive; Negative
Precuneus	R	31	24	−48	34	3.10	13	Negative
Angular gyrus	R	39	46	−64	32	3.07	45	
**OCCIPITAL**								
Inferior temporal gyrus	R	37	48	−66	−4	3.45	187	Negative
Cuneus	R	19	2	−92	24	2.95	24	
**OTHER**								
Insula	R	13	48	−24	16	3.59	272	Negative
	R	13	30	−32	26	3.37	57	Negative
	L	13	−34	−20	26	2.86	12	Negative
Putamen	R	NA	24	−2	20	3.37	37	Negative
Caudate	L	NA	−16	18	−4	3.26	68	Negative
	L	NA	−22	−26	22	3.12	44	Negative
Thalamus	L	NA	0	−20	10	3.17	31	
**Anterior cingulate**	**R**	**24**	**0**	**6**	**42**	**3.20**	^*^	Negative
		24	4	−18	44	2.90	24	Negative

*Clusters significant at an uncorrected threshold of p < 0.005, k ≥ 10 voxels*.

*Up to 3 local maxima that are at least 8 mm apart reported for each cluster*.

*BA, approximate Brodmann Area; L, Left, R, Right*.

* *Local maximum of Premotor Cortex cluster (k = 513)*.

*Negative = Age is associated with a decreased relationship between functional and structural connectivity during negative event retrieval (p < 0.005)*.

*Positive = Age is associated win an increased relationship between functional and structural connectivity during positive event retrieval (p < 0.005)*.

*Regions in bold are of theoretical importance and were interrogated further*.

**Figure 3:**
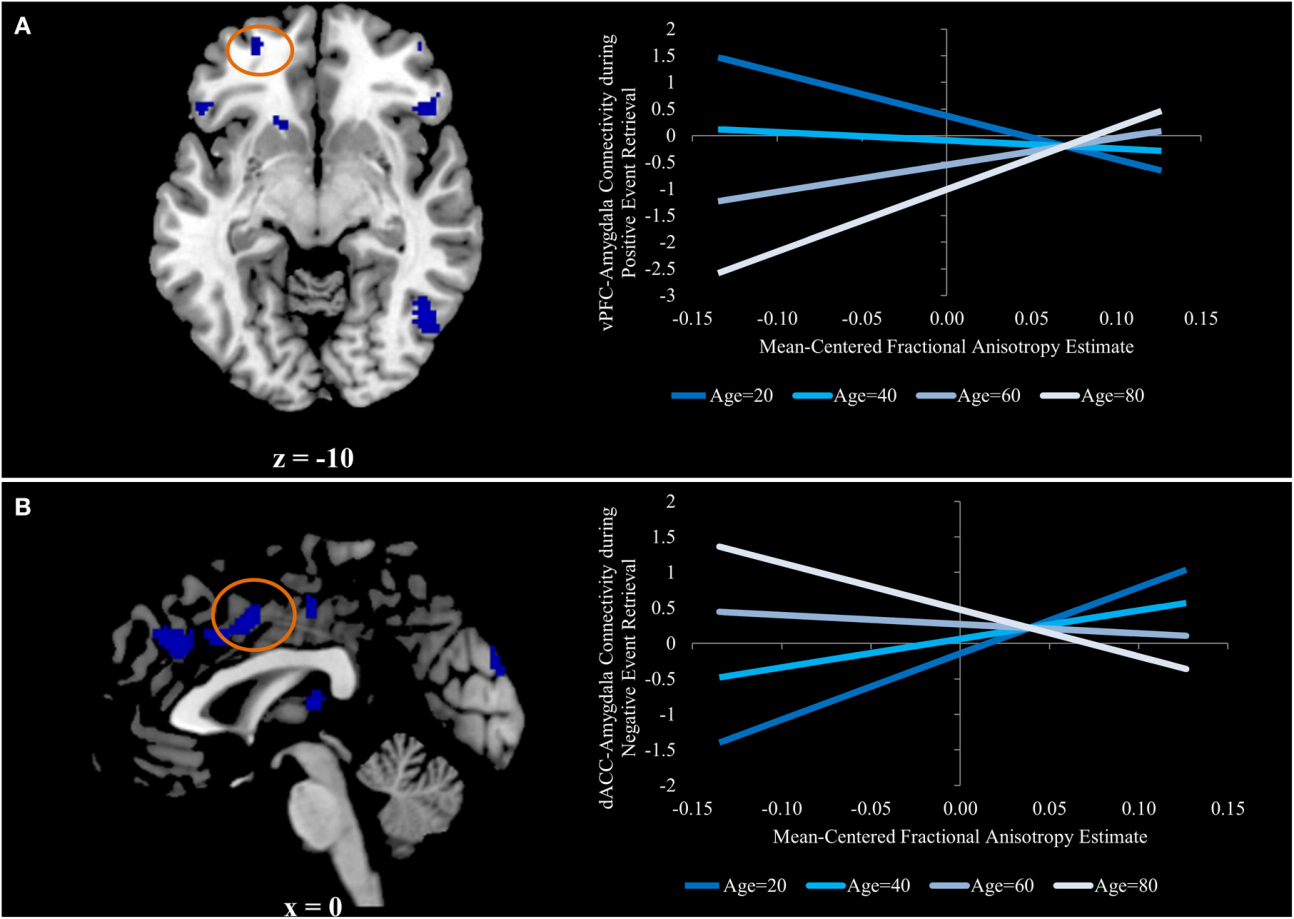
**(A)** Ventral prefrontal region in which aging is associated with an increased relation between functional and structural connectivity for positive event retrieval, but not during negative or neutral event retrieval. **(B)** Dorsal anterior cingulate region in which aging is associated with a stronger negative relation between functional and structural connectivity for negative event retrieval, but not during positive or neutral event retrieval. Graphs for both panels depict the regression equation at four representative ages (age = 20, age = 40, age =60, and age =80) to visualize the interaction.

The only two regions in which this contrast was exclusively driven by an increased relation during positive event retrieval were ventral PFC regions. The uncinate is primarily implicated in structural connections between the amygdala and ventral PFC regions (Schmahmann and Pandya, 2006; Petrides and Pandya, 2007; Lehman et al., 2011), making this pattern of particular interest. To better understand the interactive effects of age and FA on amygdala-vPFC functional connectivity, beta estimates of amygdalar functional connectivity were extracted from a 5 mm sphere surrounding a peak voxel within the vPFC (−26, 54, −10; Figure 3A). Regression analyses were generated with mean-centered age, mean-centered FA, and the interaction of age and FA as predictor variables. Because three models were generated, each was evaluated at a corrected *p*-value of *p* < 0.016 (0.05/3). The models predicting functional connectivity during neutral and negative event retrieval were not significant (*p* = 0.96 and *p* = 0.57, respectively), but the model significantly predicted functional connectivity between the amygdala and vPFC during positive event retrieval [*F*_(3, 54)_ = 5.06; *p* < 0.005]. Within this significant model, age was associated with overall declines in functional connectivity (β = −0.37, *p* < 0.005) and with increased relations between functional and structural connectivity (β = 0.33, *p* < 0.01). The effect of FA was not significant in this model (*p* = 0.78), suggesting that FA was not related to amygdala-vPFC functional connectivity at the mean of age (47 years old, or within the middle-age range). To better understand this model and the interaction, it has been plotted for four representative ages (age = 20, age = 40, age = 60, and age = 80; Figure 3A).

Finally, we re-ran the regression model with linear transformations applied to the Age variable to make each decade (i.e., 20, 30, 40, etc.) the zero-point. Re-centering a first-order coefficient such as age can facilitate the interpretation of other first-order coefficients, such as FA, in the presence of a significant interaction. These transformations preserve all other characteristics of the model (Dalal and Zicker, 2012), so the significance [*F*_(3, 54)_ = 5.06; *p* < 0.005], the effects of age (β = −0.37, *p* < 0.005), and the age-by-FA interaction (β = 0.33, *p* < 0.01) remained exactly the same. Interestingly, the effect of FA was significant when the zero point was set at 80 years old (β = 0.54, *p* < 0.05) and 70 years old (β = 0.39, *p* < 0.05), but not at any other decade (*p* = 0.1, *p* = 0.56, *p* = 0.54, and *p* = 0.17 for 60, 50, 40, and 30, respectively). Additionally, there was a trend toward a negative effect of FA on functional connectivity when the zero point was set at 20 years old (β = −0.40, *p* = 0.08).

### Effects of aging and emotion on the relation between white matter integrity and functional connectivity between the amygdala and the dorsal ACC

The results above reveal a three-way interaction in which the effects of age on the relation between structural integrity and amygdala-vPFC functional connectivity is significant during positive event retrieval, but not negative or neutral event retrieval. Such findings suggest that when older adults attempt to recall positive information, they increase their engagement of the amygdala-vPFC pathway *if* they have the structural integrity to do so.

If this is interpretation is correct, it is possible that aging would also be associated with situations where increased structural connectivity was related to negative functional connectivity, specifically in conditions where negative functional connectivity would be advantageous. Indeed, age-related decreases in functional-structural relations drove the interaction in a number of regions (denoted by “negative” in Table 2). Prior to observing the pattern (“negative” or “positive” in Table 2) in all regions, one theoretically important region, the dorsal anterior cingulate cortex (dACC), was identified as being likely to show this “negative” pattern. The dACC has been implicated in both automatic and voluntary emotion regulation, such that increased dACC activity may reduce negative emotions through regulation of regions involved in negative affect (See Phillips et al., 2008 for a review). Along this pathway, increased regulation should be revealed through more negative dACC-amygdala functional connectivity.

Beta estimates of amygdalar functional connectivity were extracted from a 5 mm sphere surrounding a peak voxel within the dACC (0, 6, 42; Figure 3B) for each emotion condition, and the regression analyses described in the previous section were run predicting these estimates of functional connectivity. As before, models were evaluated at the corrected *p*-value of *p* < 0.016. The models predicting functional connectivity during neutral and positive event retrieval were not significant (*p* = 0.94 and *p* = 0.24, respectively), but the model significantly predicted functional connectivity between the amygdala and dACC during negative event retrieval [*F*_(3,54)_ = 4.60; *p* < 0.01]. Within this significant model, age was associated with decreased relations between functional and structural connectivity [β = −0.39, *p* < 0.05). The effect of FA was not significant in this model (*p* = 0.23), suggesting that FA was not related to amygdala-dACC functional connectivity at the mean of age (47 years old, or within the middle-age range). Age was associated with a trend in which increased age was linked to numerical increases in functional connectivity (*p* = 0.053)^1^. The effect of FA on functional connectivity has been plotted in Figure 3 for representative ages along the age range (specifically, each decade). The age-transformed regression analyses revealed a significant negative relation between FA and amygdala-dACC functional connectivity when the zero point was set at 80 years old (β = −0.45, *p* < 0.05) and a significant positive relation when it was set at 20 (β = 0.67, *p* < 0.005), 30 (β = 0.48, *p* < 0.001), and 40 (β = 0.30, *p* < 0.05). However the relation was insignificant in the middle-age range (*p* = 0.39, *p* = 0.57, and *p* = 0.13 for 50, 60, and 70, respectively). To better understand this model and the interaction, it has been plotted for four representative ages (age = 20, age = 40, age = 60, and age = 80; Figure 3B).

## Discussion

The present study is the first to demonstrate that participant characteristics (such as age) and task characteristics (such as memory valence) can interact to influence the relation between functional and structural connectivity. Aging was associated with increased relations between measures of structural integrity and functional connectivity such that, in older adults only, increased structural integrity was associated with more positive functional connectivity between the amgydala and the ventral PFC during positive event retrieval. Furthermore, older adults showed greater negative relations between structural integrity and functional connectivity with the amygdala during negative event retrieval in numerous regions including the dACC. This pattern is consistent with the role of the dACC in emotion regulation. These results demonstrate the insights that can be gained by considering structural and functional connectivity together.

### Measuring structural and functional connectivity: the importance of examining the interaction

Although aging is typically associated with overall decreases in structural integrity (e.g., O'Sullivan et al., 2001; Pfefferbaum and Sullivan, 2003; Madden et al., 2004), the current study found that aging was not associated with changes in structural integrity along the uncinate fasciculus. This finding is consistent with another recent study showing no age-related differences in FA in the left UF (Davis et al., 2009), and may reflect the fact that aging does not impact all tracts equally (Madden et al., 2012). Such null results, on their own, may suggest that aging does not influence connections between the vPFC and the amygdala. However, by examining the effect of aging on the interaction of these connectivity measures, the current study identified an important age-related effect that would have otherwise gone unidentified. Specifically, the current study demonstrated that older adults exhibit increased functional connectivity of vPFC and amygdala regions if they have the structural pathways to allow these connections. Importantly, age is only one example of a population characteristic that may influence the interaction between structural and functional connectivity even in the absence of a direct relationship with either connectivity measure. These interactions should be considered in all connectivity studies, as they are critical to understanding (a) the relation between structural and functional connectivity or, more specifically, the situational parameters under which structural connectivity constrains communication between neural regions, and (b) the cognitive mechanisms that might increase or decrease reliance on a particular anatomical connection (see below).

As with all null results, it is important to note that true differences (e.g., in structural connectivity of the uncinate fasciculus with age) may exist that were not successfully identified in the current study, either from a lack of power or the selection of an incorrect measure. Whether or not aging influences structural integrity along the uncinate fasciculus, the current study demonstrates that the interaction may be identified in circumstances when the age effects of structural integrity are more subtle. As such, the benefits of such analyses are still clear.

### Effects of aging: potential cognitive mechanisms

An age-by-FA interaction was identified in the current study such that the effect of structural integrity on functional connectivity differed as a function of age, with functional connectivity relying on structural integrity more with increasing age. Importantly, this interaction varied as a function of emotional valence, suggesting that the age-related changes may be associated with distinct cognitive processes.

The effect of structural integrity on functional connectivity between the amygdala and vPFC was only present during retrieval of positive events, with no apparent effects during negative or neutral events, and it was driven by a strong positive relationship in older adults only. It has been suggested that memory impairments in older adults can be mitigated by the presence of emotional arousal (e.g., Kensinger, 2009), particularly when the information is of positive valence (e.g., Charles et al., 2003). As such, it is possible that this interaction reflects cognitive mechanisms unique to retrieval of *positive* information. One primary explanation for differences in how older adults interact with positive and negative information is that older adults are motivated to regulate their emotional state to a greater extent than young adults due to changes in priorities and goal states during retrieval (Mather and Carstensen, 2005). The left vPFC has been implicated in emotion regulation during memory retrieval (Holland and Kensinger, 2013), guiding retrieval of emotional details and either increasing or decreasing neural activity in limbic regions (such as the amygdala) associated with emotional responses (Ochsner and Gross, 2005). Thus, age-related changes in emotional processing may cause older adults to recruit vPFC regions to increase amygdala activity, and therefore emotional reactions, during positive event retrieval.

If the age-related increases in relations between structural and functional connectivity are driven by increased emotion regulation, it holds that there also should be PFC regions whose activity is related to lowered activity in the amygdala during negative event retrieval (i.e., negative functional connectivity between prefrontal regions and the amygdala during negative event retrieval) as a function of structural integrity. The current study identified such a relationship in the dACC, such that aging affected the extent to which structural integrity predicted stronger negative functional connectivity with the amygdala during negative event retrieval. It has been suggested that the dACC exerts top-down control on the limbic system in both voluntary and automatic regulation (Phillips et al., 2008). In addition, a recent review identified a similar dACC cluster (0, 12, 42, compared to our cluster at 0, 6, 42) as representing the intersection of negative affect, pain, and cognitive control (Shackman et al., 2011). The results of the current study suggest that aging may affect dACC down-regulation of amygdalar activity during negative event retrieval, suggesting that the interactions identified in the current study may reflect age-related changes in emotion regulation.

The finding that age and emotion interacted to modulate the relation between functional and structural connectivity is consistent with behavioral studies showing that older adults exhibit enhanced positivity in their memory retrieval when sufficient cognitive resources are available (Mather and Knight, 2005). In other words, this prior study revealed that aging may be associated with an increased reliance on cognitive control processes to regulate emotions during memory. The current study extends this research by revealing that there are valence-dependent patterns of functional connectivity that differ both as a function of age and as a function of structural connectivity.

## Conclusions

The current study was the first to demonstrate how participant characteristics (such as age) and task characteristics (such as memory valence) may interact to influence the relation between functional and structural connectivity. Importantly, age did not affect structural integrity of the tract evaluated in the current study, demonstrating that considering structural and functional connectivity together may be critical to understanding the effects of a particular variable (in this case, age) on connectivity between two regions. Here, the interactive effect of age and FA on functional connectivity between the PFC and amygdala is consistent with theories that older adults may engage regulatory strategies during the retrieval of emotional information if they have the structural pathways to allow them to do so.

### Conflict of interest statement

The authors declare that the research was conducted in the absence of any commercial or financial relationships that could be construed as a potential conflict of interest.

## Footnotes

^1^Fractional anisotropy is only one of many measures of structural integrity. To better characterize the relation between functional connectivity and structural integrity, we ran the regression analyses described above with three additional measures of structural integrity: mean diffusivity, axial diffusivity, and radial diffusivity. When using a false discovery rate correction for 24 comparisons (2 anatomical regions × 3 valence conditions × 4 structural connectivity measures), the effects of FA reported in the manuscript retained significance. Measures of radial diffusivity and mean diffusivity exhibited the same patterns as measures of FA, although the corrected significance for the model including mean diffusivity to predict negative event connectivity in the dmPFC was only a trend (*p* = 0.056). Interestingly, the models including axial diffusivity to predict connectivity were insignificant, although there was a trend for positive event connectivity in the vPFC (*p* = 0.08). These findings are consistent with prior studies showing that effects of aging are greater for radial relative to axial diffusivity (Davis et al., 2009).
